# When less is more: single selfhood-related cues elicit higher selfhood ratings than multiple cues

**DOI:** 10.3389/fcogn.2026.1815906

**Published:** 2026-06-24

**Authors:** Jan Pohl, Kristina Nikolovska, Francesco Maurelli, Arvid Kappas, Bernhard Hommel

**Affiliations:** 1 Faculty of Psychology, Dresden University of Technology, Dresden, Germany; 2 University Clinic for Psychosomatic Medicine and Psychotherapy, Otto von Guericke University Magdeburg, Magdeburg, Germany; 3 School of Computer Science and Engineering, Constructor University, Bremen, Germany; 4 School of Business, Social and Decision Sciences, Constructor University, Bremen, Germany; 5 Department of Psychology, Shandong Normal University, Jinan, China

**Keywords:** agency, anthropomorphism, attribution, bootstrapping, human-robot interaction, mind, non-humanoid robot, self

## Abstract

**Introduction:**

In everyday social cognition, people reliably tend to anthropomorphize non-human agents or ascribe to them features associated with selfhood. In prior research, we showed that individuals often (over-)generalize from a single behavioral indicator of selfhood, such as learning ability or attentional sharing, to the assumption that other, unobserved indicators of selfhood are also present. This suggests that even minimal cues may be sufficient to activate a broader selfhood concept and its associated implications.

**Method:**

Here, we tested the prediction that single selfhood cues are as effective as multiple selfhood cues in eliciting selfhood- attributions to an artificial agent. In three experiments, we compared selfhood ratings for a robot exhibiting behavioral cues of efficiency, learning sensitivity, and equifinality with ratings for a robot exhibiting only one of these cues.

**Results:**

Contrary to our expectations, participants did not attribute the same degree of selfhood to both robots. They attributed more selfhood to the single-cue robot in Experiments 1 (single efficiency cue) and 3 (single equifinality cue), whereas in Experiment 2 (single learning cue), the multiple-cue robot received higher ratings on the manipulated characteristics as well as on context sensitivity (results for selfhood-attribution were negligible).

**Discussion:**

In sum, we conclude that a single selfhood cue tends to elicit stronger selfhood-attributions overall than multiple cues. We discuss this finding in relation to the literature on cognitive bootstrapping (i.e., developing new and complex knowledge from a limited starting set), concluding that consistently applying a single skill to an apparent problem may reflect more human-like behavior.

## 1 Introduction

In everyday contexts, people readily extend elements of human selfhood to non-human entities and interact with them accordingly. For instance, technological systems are often treated similarly to humans, as described by the Media Equation (Nass et al., 1999; Reeves and Nass, 1996). Likewise, individuals attribute personality traits to dogs (Gosling et al., 2003) and infer intentionality, even in simple cases such as automatic doors (Ju and Takayama, 2009) or abstract geometric shapes (Heider and Simmel, 1944). Despite ongoing debate within the scientific literature—where neither a unified definition of the self exists (Leary, 2004) nor consensus on its very existence (e.g., Bennett and Hacker, 2022; Giles, 1993)—laypeople routinely rely on intuitive, or “naïve”, notions of selfhood in everyday reasoning. Such notions play a central role in how behavior is interpreted, as people commonly explain others' actions by appealing to internal mental states and selfhood-related concepts, for example, through frameworks like Theory of Mind (e.g., Frith and Frith, 2005) or by adopting an intentional stance (Dennett, 1989). As we have argued before (Pohl et al., 2025, 2026), this pattern of reasoning raises a key question: by what processes do individuals come to ascribe selfhood to an observed agent, and which cues or characteristics facilitate these attributions?

### 1.1 Self as a social role

In this study, we are interested in the subjective concept of the self as it is used in everyday life to describe others. Conversely, we were not interested in how “the self” might be defined or function in an individual, but how this concept is used by people to describe other agents. In the field of emotion research, a comparable approach has been developed by Averill (1980), who characterized emotions as “transient social roles.” He began to define emotions in terms of their “aboutness,” or the object or situation that elicits the emotion. For instance, disgust might be about the rotten food in our refrigerator, and anger might be about the person blocking the door to our home. Moreover, Averill observed that emotions are personal evaluations of a given situation. Therefore, emotions are inherently passive; they do not act on their own. He concurred with Schachter's (1971) assertion that a single arousal state may elicit distinct emotional labels based on situational cues. However, Averill posited that individuals do not merely categorize internal states or behaviors. Instead, individuals assume a relationship to the object of their emotion, which is most often another person. Consequently, he views emotions as a social role. Moreover, he proposed that both the meaning of emotions and the corresponding behavior are learned by individuals. This is also true for the concept of the self, as evidenced by the extensive literature on Theory of Mind (see e.g., Frith and Frith, 2005). Children demonstrate growth in their ability to attribute states and beliefs to others, and their concept of selfhood undergoes a developmental trajectory.

Understanding the self, analogous to Averill's (1980) understanding of emotions, merely as a passive social role poses no essential conflicts with many of the current notions of the self. For instance, the scientific concept of the narrative self (Schechtman, 2011), refers to a self-image comprising stories *about* oneself in the past or future. (Giles 1993) described the self similarly not as a persisting self but as a self-image that is constantly changing. Hence, in these theories, the self can be understood as a passive appraisal of oneself most of the time, in social situations. Moreover, even the conscious experience of selfhood in the moment, what has been described as the minimal self (Gallagher, 2000), can be explained as a passive self-reference, as has been proposed by Metzinger (2004). Metzinger's theory posits that the brain constructs a model of oneself embedded into the physical world, including one's own body, and that with the constant interaction of sensory inputs with this self-representation, the model becomes transparent: The brain takes the perspective of the model, because the model is not perceived as such (see Limanowski and Blankenburg, 2013 for a more in-depth review on this topic).

A further similarity between the fields of emotion and selfhood is the multitude of theories that have been proposed. For example, among proponents of a basic emotion approach, there is no consensus on the number or identity of basic emotions (see e.g., Kowalska and Wróbel, 2020; Ortony, 2022). From a constructivist perspective, this is not a problematic issue. Averill, for instance, does not propose that some emotions are more fundamental than others. Rather, there are as many different emotional labels as are required by individuals—including scientists at work—within their social context. While emotional roles may be derived from and incorporate physiological aspects, Averill asserts that “the meaning of the emotion—its functional significance—is to be found primarily within the sociocultural system” (1980, p. 337). From the perspective of selfhood, we encounter an even more extensive discourse, comprising a multitude of explanatory approaches to the self. This has prompted, for example, Gallagher (2013) to put forth a Pattern Theory of Self, merging common aspects of different theories. The various approaches to the self may, however, be seen to constitute the social roles that the self plays in human interaction, just as the multiplicity of emotional labels helps to navigate specific interactions. In light of this comparison, it is at least conceivable that an objective self might exist, which inspired these definitions. Nonetheless, viewing the self as a social construct suggests that there is no need for an inherent, fundamental self and that the various theories may simply reflect different functions of explaining human behavior. Hommel (2019) described a similar approach to the self in the form of a loose feature network. People may represent themselves and others by a certain number of feature codes, which may differ across people or situations, and change over time. For each individual, they form an overarching concept of the self, filled or connected with different (social) roles. Overall, it seems sufficient to approach selfhood from a constructivist perspective.

### 1.2 Attribution theory

**Figure 1:**
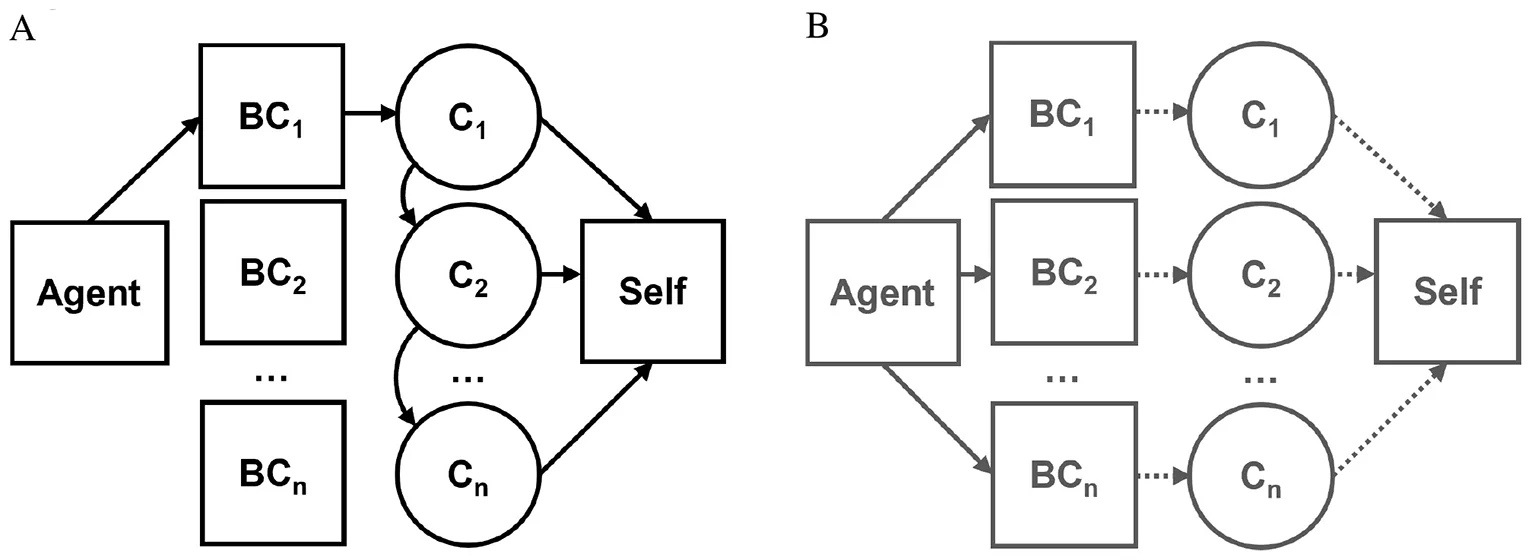
When less is more. Contrary to our expectation, the over-generalizing **(A)** from an agent's behavioral cues (BC) for a single characteristic (C) to other characteristics leads to stronger selfhood ratings as compared to an agent that is indeed exhibiting cues for multiple characteristics **(B)**.

If selfhood is a socially learned interpretive construct, then attributing a self to others is necessarily an inferential process based on limited observable cues. Attributing selfhood, thus, can be conceptualized as a general attribution process in which observers project internal causes—such as agency, intention, or experience—onto observed behavior. Attribution theory provides a natural theoretical framework for formalizing this process, because it explicitly models how people explain others' actions under conditions of limited information and systematic bias. Heider's (1958) attribution theory focuses on the general phenomenon of attributing naïve concepts to other agents. In his view, people are not aware that their perception of the world is often based on insufficient perceptual information and, thus, is open to subjective biases. Rather, people seem to assert that their notion of the world is objective and starts to form theories about others' behavior. These theories often falsely assume that actions are a result of personal causes like agency and intention, as indicated by the fundamental attribution error (Ross, 1977). Along the same line, Brunswik's (1955) lens model of perception does not assume that people have direct access to the objective state of the world or to a perfectly valid representation thereof. Rather, people are assumed to interpret the world based on proximal (subjective) information triggered by the distal state of the world. Applied to selfhood, this would mean that in order to derive a judgement on whether an observed agent has a self, people need to utilize behavioral, *distal* cues that the agent exhibits, which then trigger the *proximal* perception of characteristics associated with selfhood. In our account (see Figure 1A), this process involves distinct levels of analysis: it begins with an immediate perceptual judgment of distal behavioral cues, which triggers an inferential attribution of internal characteristics, ultimately culminating in a *post-hoc* explanation for the agent's behavior in the sense of Heider's “naïve psychology.”

### 1.3 The naïve self

Combining the constructivist approach with these assumptions from attribution theory, we propose that selfhood functions as a “transient social role” that observers assign to agents to navigate social interactions. In this view, “selfhood” is not an internal property to be discovered, but a social-cognitive label used to organize perceptions of observable behavior related to attributed dimensions such as agency, experience, cognitive functioning, and sociality. In this sense, selfhood serves as an umbrella term combining questions from mind-attribution, research on anthropomorphism, and social attribution. This explains why our operationalization relies on a broad range of established scales: the mind-attribution Scale (MAS) (Bigman and Gray, 2018), the Godspeed Scale (GS) (Bartneck et al., 2009), and the Robotic Social Attributes Scale (RoSAS) (Carpinella et al., 2017). Specifically, the *agency* and *experience* subscales of the MAS were included, as these dimensions are central to common philosophical accounts of selfhood (Gallagher, 2000, 2012; Woolf, 1997) and have been highlighted in key empirical research on mind perception (Gray et al., 2007), while also directly informing judgments about capacities for intentional action and subjective experience. Furthermore, the GS was used to assess broader perceptual dimensions, including *perceived intelligence*—reflecting attributions of higher cognitive abilities noted in prior work (e.g., Eddy et al., 1993; Ju and Takayama, 2009)—as well as *animacy*, which partially overlaps with agency (Westfall, 2023), and *anthropomorphism*, a widely used as an indicator of perceived human-likeness. To further account for social evaluations, the RoSAS was included, measuring perceived *warmth, competence*, and *discomfort*, given the established importance of social interaction in the development and functioning of the self (Cooley, 1998; Mead, 1913).

These scales do not represent disparate constructs in the view of the observer but rather as indicators of the same underlying role-attribution (as we already argued previously; Pohl et al., 2025). As such, the conceptualization of selfhood-attribution presented here is inherently overlaps with established approaches—such as Gray et al.'s (2007) mind perception or Epley et al.'s (2007) theory of anthropomorphism—it is not intended to introduce a new, isolated psychological construct. Rather, it extends on these frameworks by synthesizing them into a holistic account of how observers interpret the internal states of other agents. Moreover, by combining established questionnaires into a unified framework, which is rooted in Heider's (1958) attribution theory and Brunswik's (1955) probabilistic functionalism (as described in the previous section), we attempted to capture a broader social-cognitive inference than isolated trait attributions alone.

### 1.4 Aim of the present study

Building upon our previous empirical studies (Pohl et al., 2026, 2025), we used a Brunswikian framework to isolate specific behavioral characteristics that cue the perception of a “self” in artificial agents. Our initial experiments utilized simple, non-humanoid robotic platforms—reminiscent of Braitenberg's (1986) “vehicles”—to investigate how minimal movement patterns influence observers' attributions. By manipulating these agents to exhibit either the presence (critical) or absence (control) of certain core characteristics, we evaluated whether these minimal distal cues could trigger a shift in proximal perception toward selfhood-attribution. Participants viewed videos of these robots navigating obstacle-filled environments and provided ratings on a multidimensional suite of (sub-)scales—including measures of agency, experience, and intelligence—while we remained intentionally agnostic regarding the ontological status of a “true self” in favor of capturing the breadth of subjective perception.

Our prior investigations spanned a variety of behavioral characteristics, ranging from non-social cues like equifinality and learning sensitivity to social cues, such as joint attention and helping behavior. While participants reliably identified the intended manipulations, a consistent and unexpected pattern of over-generalization emerged: most of the investigated characteristics boosted the scores of other, non-manipulated, characteristics as well (e.g., a more efficient robot was also perceived to show more causal behavior, even though the latter did not vary between conditions). This cross-characteristic boost suggested that the attribution process does not function as a series of isolated 1:1 mappings as a strict lens model might predict. Instead, it appears that a single salient cue acts as a gateway, activating a broader conceptual network of selfhood—a phenomenon we have termed the *Pars-Pro-Toto* account. In this account, even minimal evidence for one characteristic provides the cognitive foundation for observers to “fill in” a complete, agentic internal model (see Figure 1A).

Based on this account, we hypothesized that presenting participants with a single cue should not elicit significantly fewer selfhood-attributions than presenting them with multiple cues (see Figure 1B), as participants should over-generalize from this single cue to others anyway. Accordingly, we equipped one robot with a single behavioral cue and another one with three behavioral cues—all cues were taken from the set of non-social cues that we previously found to induce over-generalization (Pohl et al., 2026)—and had participants rate both robots like the critical and control robots in our previous studies. More specifically, we adopted three cues from our previous study of non-social characteristics to equip our present multiple-cue robot: efficiency, learning sensitivity, and equifinality. We then designed three experiments comparing this multiple-cue robot with a single-cue robot that exhibited only a single cue: efficiency in Experiment 1, learning sensitivity in Experiment 2, and equifinality in Experiment 3. The key question was whether the multiple-cue robot would elicit higher selfhood ratings than the single-cue robot, as a simple (cumulative) Brunswikian approach would suggest, or whether the ratings for the two robots would be comparable, as our Pars-Pro-Toto account implies.

## 2 Materials and methods

The methods, as described below, were generally consistent with those in Pohl et al. (2025). We also assessed participants' prior experience with robots (Schaefer, 2016), at the end of the session. Scales and other materials can be found at the Open Science Framework (OSF) (Foster and Deardorff, 2017) under *osf.io/v62sg*.

### 2.1 Participants

Participants were recruited from the UK via Prolific. Recruitment continued until 80 valid data sets were collected for each of the three experiments. Participants received £4 for completing the study. In total 253 participants took part in the study, and data collection continued until 80 valid data sets per experiment were collected, resulting in 240 included participants (see Table 1 for demographic data). Participants were excluded from the analysis if they reported considerable technical difficulties (e.g., stuttering of the videos, which resulted in the exclusion of *n* = 6 or 2% of the total number of recruited participants) or if they failed the attention check (as described in the *Procedure* section below, which resulted in the exclusion of *n* = 7 or 3% of all recruited participants).

**Table 1:** Demographic overview of participants per experiment.

		Age		Gender (in %)		PERQ^a^	
Exp.	*n*	*M*	*SD*	Female	Male	*M*	*SD*
1	80	34.85	12.08	51	49	2.02	0.87
2	80	38.91	12.81	48	52	2.26	0.98
3	80	36.96	11.03	51	49	2.20	0.87

^a^Data from Item 4 of the Prior Experience with Robots Questionnaire (PERQ), which is a self-reported knowledge estimate about robots and their capabilities on a scale of 1–5.

### 2.2 Stimuli

Stimuli consisted of videos captured using a Panasonic HC-V380EG-K camera in our laboratory. They were shot from above at an angle, showing a “Duckie Mobile Bot” (Paull et al., 2017) robot identified by either a white triangle or square, and a second unmarked robot. The marked robots were the robots of interest to be rated by the participants. We also utilized white and black cardboard cubes as obstacles in all experiments (the stimuli can also be found in the online material at *osf.io/v62sg*).

The robots of interest were remotely controlled to cue either all three characteristics (*multiple* cues) or only one of them (*single* cues). Each experiment presented an equal number of stimuli for the multiple- and single-cue conditions (5 each). Additionally, there was always a set of videos showing the multiple cues robot and the single-cue robot in the same setup. Stimuli suggesting multiple characteristics were the same for all experiments. All stimuli showed a complex arrangement of mostly white cubes, which were stationary, and some movable black cubes. Furthermore, there were always two “goal” positions marked with white tape, one of which looked like an “X” while the other resembled a serif “I.” Although the stimuli were matched with respect to the scenario content, they differed in duration due to the multi-experiment design.

*Across all experiments*, the videos presenting multiple cues (between 10s and 19s) showed a robot that always ended its movement on the same goal (“I”) to suggest equifinality. Simultaneously, it chose the shortest path to demonstrate efficient behavior. In the process of reaching the goal, the robot tried to push the black and white cubes (creating shorter paths). To cue learning sensitivity, the number of white cubes pushed before it successfully pushed a black cube was decreasing both within the videos but also between the videos (e.g., in the first video, the robot pushed the first 3 white cubes before a black one and consequently, at a later part in the same video, only 1 white cube, while in the last video, the robot no longer tried to push the white cubes but went directly to push the black one).

In *Experiment 1*, the stimuli for efficient behavior (between 6s and 10s) showed the robot always choosing the shortest available path to the closest goal, either “X” or “I” and, thus, not suggesting equifinality. Furthermore, the robot showed no attempt to push the cubes to create even shorter paths, thus missing any indications of learning behavior.

For *Experiment 2*, in the stimuli suggesting only the presence of learning sensitivity (between 14s and 29s) the robot's behavior of pushing cubes to create new paths looked similar to the robot showing multiple cues. While the number of attempts to push white cubes decreased within and between videos in the same way, the learning robot alternated between the two possible goal locations and never reached them via the shortest possible path, suggesting the absence of equifinality or efficiency.

In *Experiment 3*, the stimuli suggesting only the presence of equifinality (between 11s and 19s) showed the robot always moving toward the same goal (“I”). Just as the efficient robot, this robot never tried to push the cubes, thus, not showing indications for learning sensitivity and requiring the robot to take longer paths than the multiple cues robot, making it less efficient.

### 2.3 Implementation

The study was run as in-browser experiments that participants could participate from home. The experiments were programmed with jsPsych 7.3 (de Leeuw et al., 2023) and hosted on a university server with JATOS 3.7.4 (Lange et al., 2015).

### 2.4 Procedure

The experiment commenced with a consent form and instructions (with approval from Constructor University's ethics committee). The experiment then continued with the presentation of stimuli. Multiple and single cue stimuli were presented in alternation. The videos of one condition were consistently displayed either on the left or the right of the window, while the mapping of the condition and alignment was systematically counterbalanced. Following the stimulus presentation, participants completed several questionnaires. In the first four questionnaires, they rated each robot separately, while the subsequent questionnaire asked about the participants themselves. At the end of each experiment, participants were debriefed and asked what they thought the research question was whether they experienced any technical difficulties with the study. Those participants who reported major problems (e.g., faulty stimulus presentation) were removed from the analysis (resulting in *n* = 6 exclusions, or 2% of all recruited participants).

The study used a manipulation check scale (MCS) with three items for each characteristic of interest^1^. E.g., for the characteristic causality, the items “It was able to affect its environment”, “It seemed to intentionally interact with its environment” and “It appeared to desire to modify its environment,” were used. The items were composed to range from simple descriptions devoid of selfhood-related states to descriptions referencing intentions similar to the answers of participants in Heider and Simmel's (1944) study (see the OSF repository for the complete questionnaire).

For accessing selfhood-attribution, three already established questionnaires were used: The subscales *agency* and *experience* of the Mind-attribution Scale (MAS) (Bigman and Gray, 2018), the Godspeed Scale (GS) (Bartneck et al., 2009), as well as the Robotic Social Attributes Scale (RoSAS) (Carpinella et al., 2017). The scales were implemented as continuous scales, ranging from 0 to 100, with sliders marked only at the extremes: 0 and 100 for the MCS; “Disagree” and “Agree” for the MAS; negative and positive items for the GS and RoSAS. The change from Likert to continuous scales was made, whereas the choice of scale has no effect within a test (e.g., García-Pérez, 2024; Kuhlmann et al., 2017), all questionnaires were presented twice and had continuous sliders, thus discouraged responses based on memory of prior responses. An attention check item asking whether the robot was able to move, supplemented the MAS. As the robots consistently moved in the videos, any participant who moved the response slider toward “Disagree” was excluded from the analysis (resulting in *n* = 7 exclusions, or 3% of all recruited participants). The MCS was completed by all participants first, and the order of MAS, GS, and RoSAS was counterbalanced, with the MAS always in the middle to ensure that the attention check was placed at the same point in the study flow. Furthermore, we used as exploratory measures the Prior Experience with Robots Questionnaire (PERQ) (Schaefer, 2016) to gather additional information about our participants. The PERQ provides information about participants' knowledge about robots and their capabilities. To avoid participants being influenced by these questionnaires, they were collected after the main experiment.

### 2.5 Data analysis

All statistical analyses were conducted using the statistics software *R* (Version 4.2.2., R Core Team, 2022). The analysis code is published in the OSF project. For each experiment, we first analyzed the data of the behavioral characteristics. Here ratings were aggregated by participant, cues (*multiple* vs. *single*) and characteristic using the mean. Since our hypothesis was that the robots would not be perceived differently, we calculated Bayes factors. This way, the analysis would not only be able to reveal missing evidence for an alternative hypothesis but also potential evidence in favor of our null-hypothesis (see e.g, Tendeiro and Kiers, 2019). This was done using the formula “*Rating*~Characteristic*Cues” as used in the R package *Bayesfactor* (Morey et al., 2026) to compare all main effects and the interaction against the null model. For comparability with our previous studies conducted by Pohl and colleagues, we also conducted a two-way ANOVA with the within-participant factors of cues (*multiple* vs. *single*) and characteristic (*causality, speed, equifinality, efficiency, learning sensitivity*, and *context sensitivity*). If there was at least positive evidence [as defined in Kass and Raftery (1995)] for an effect of the interaction, we conducted *post-hoc* paired *t*-tests for cue-presence grouped by characteristic.

Next, in cases of a significant interaction between cues and characteristic, we conducted a separate analysis of the selfhood-attribution data for those experiments. For this analysis, we categorized into subscales rather than the overall questionnaires. The data from the MAS was therefore categorized into either *agency* or *experience*, while each rating from the GS was categorized as *animacy, anthropomorphism, likability, perceived intelligence*, or *perceived safety* and RoSAS items were categorized as *warmth, competence* or *discomfort*. Ratings were then aggregated by participant, cues, and subscale using the mean. Since our hypothesis was that there would be no significant difference in selfhood-attribution between the multiple and single cue robots, we calculated Bayes factors with the formula “*Rating*~Subscale*Cues.” Furthermore, for comparability with our previous studies, we calculated a two-way ANOVA with cues (*multiple* vs. *single*) and subscales (*agency, experience, anthropomorphism, animacy, likeability, intelligence, safety, warmth, competence*, and *discomfort*) as within-participant factors. If there was at least positive evidence for an effect of the interaction, we conducted *post-hoc* paired *t*-tests for cue-presence grouped by subscale. While we included *likeability* and *perceived safety* in the analysis, we did not consider these subscales as significant contributors to assessing selfhood-attribution, rather than scales of interest in the context of social interaction or in general.

## 3 Results

### 3.1 Experiment 1: multiple cues vs. efficient behavior

**Figure 2:**
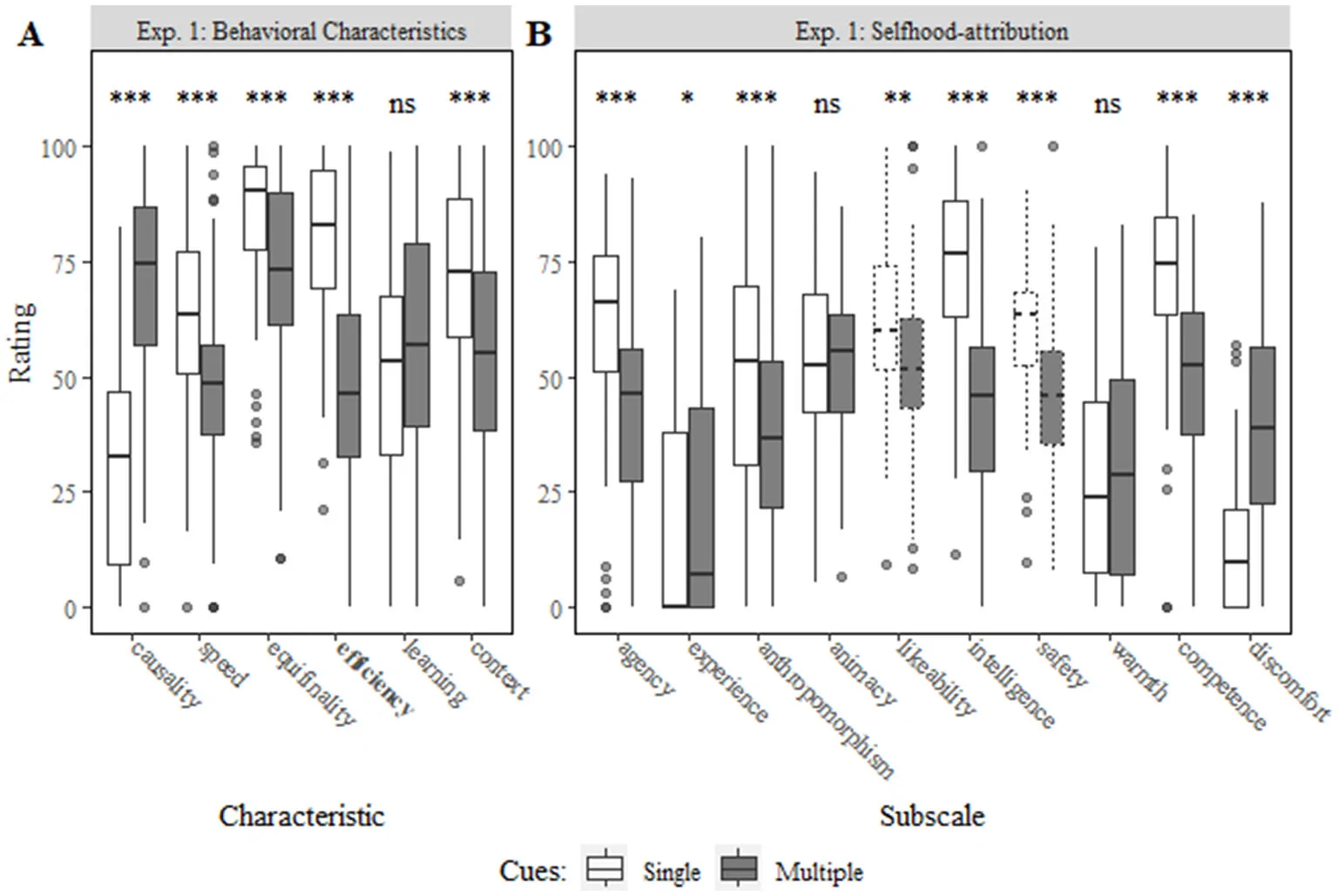
Results of experiment 1 (multiple cues vs. efficiency). **(A)** Results for the behavioral characteristics. **(B)** Results for the selfhood-attribution. Barplots printed with dotted lines are not considered critical for selfhood-attribution. Significance codes: *p* < 0.050*, *p* < 0.010**, *p* < 0.001***.

For the behavioral characteristics, the Bayesian analysis revealed substantial evidence for a main effect of cues (*BF*_10_ = 4.85, ± 0%) and decisive evidence for both the main effect of characteristic (*BF*_10_ > 100, ± 0%) and, more interestingly, the interaction (*BF*_10_ > 100, ± 0%). Consistent with these results, the ANOVA revealed a significant main effect of characteristic [*F*(4.15, 328.18) = 54.00, *p* < 0.001, η^2^ = 0.14, 95%–CI [0.07, 0.21]] and a significant interaction [*F*(4.04, 319.37) = 61.48, *p* < 0.001, η^2^ = 0.20, 95%–CI [0.12, 0.27]]. The *Post-hoc* paired *t*-tests showed significant differences for all characteristics (all *p*-values < 0.001) except learning sensitivity [*t*(79) = 1.07, *p* = 0.289] with the robot in the single condition receiving higher ratings for all characteristics except causality (see Figure 2A).

The Bayesian analysis of the selfhood-attribution data revealed decisive evidence for both main effects and the interaction, as compared to the null model (all *BF*_10_ > 100, ± 0%). Consistent with the above results, the ANOVA showed significant main effects of cues [*F*(1, 79) = 19.92, *p* < 0.001, η^2^ = 0.04, 95%–CI [0.00, 0.12]] and subscale [*F*(5, 395.06) = 121.33, *p* < 0.001, η^2^ = 0.36, 95%–CI [0.28, 0.42]], and a significant interaction [*F*(4.35, 343.49) = 47.89, *p* < 0.001, η^2^ = 0.13, 95%–CI [0.07, 0.19]]. *Post-hoc* paired *t*-tests further revealed significant differences for all subscales except animacy [*t*(79) = 0.19, *p* = 0.847] and warmth [*t*(79) = 1.36, *p* = 0.178] (all other *p*-values < = 0.010). Ratings were higher for the robot in the single condition on all subscales with significant differences except experience and discomfort (see Figure 2B).

### 3.2 Experiment 2: multiple cues vs. learning sensitivity

**Figure 3:**
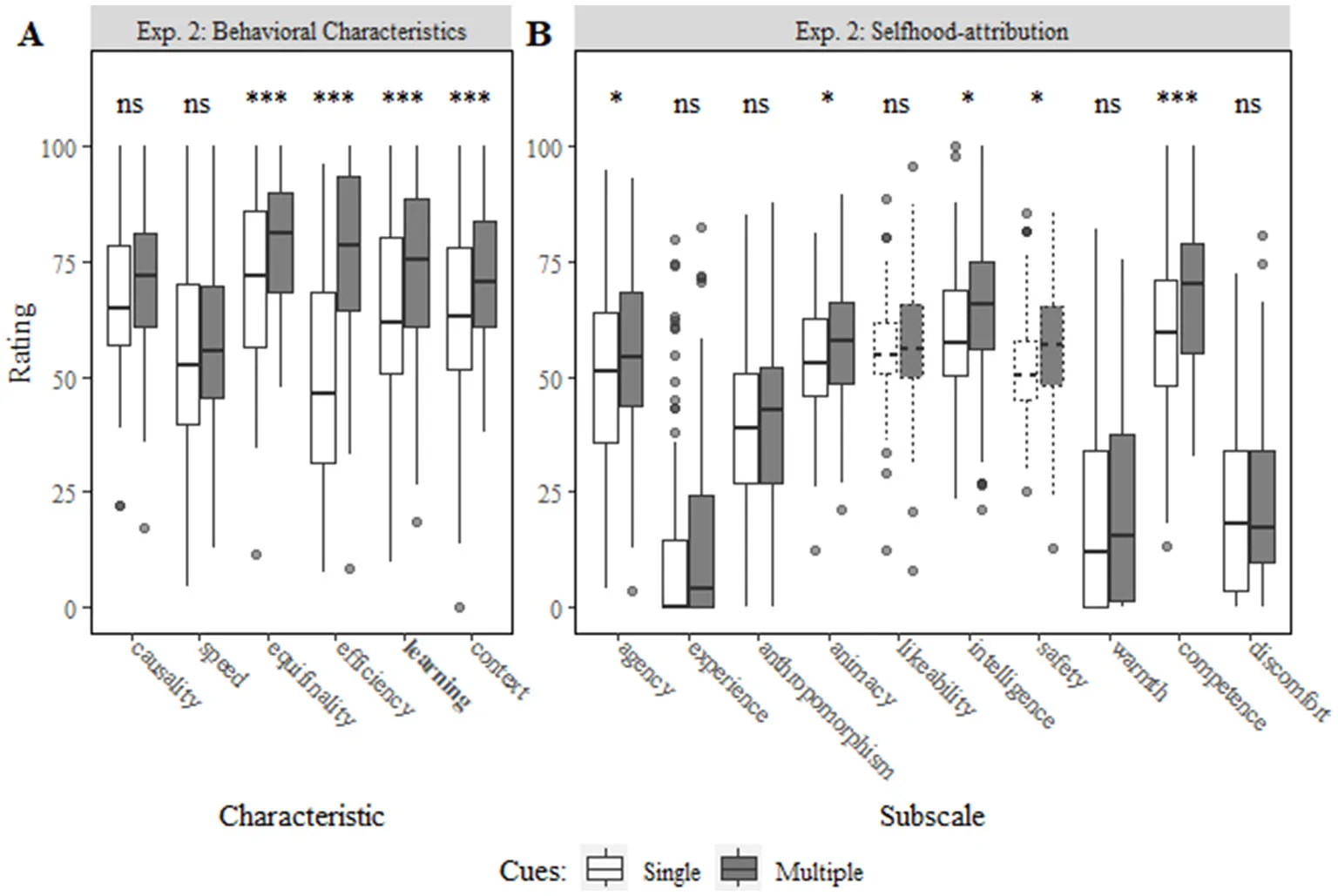
Results of experiment 2 (multiple cues vs. equifinality). **(A)** Results for the behavioral characteristics. **(B)** Results for the selfhood-attribution. Barplots printed with dotted lines are not considered critical for selfhood-attribution. Note, for the selfhood-attribution that while the frequentist ANOVA showed a significant interaction, the effect size was negligible (< 0.01), and the Bayesian analysis revealed decisive evidence against the interaction. Significance codes: *p* < 0.050*, *p* < 0.010**, *p* < 0.001***.

For the behavioral characteristics, the Bayesian analysis revealed decisive evidence for both main effects (both *BF*_10_ > 100, ±0%) and, more interestingly, the interaction (*BF*_10_ > 100, ±0%). Consistent with these results, the ANOVA showed significant main effects of cues [*F*(1, 79) = 34.62, *p* < 0.001, η^2^ = 0.06, 95%-CI [0.00, 0.16]] characteristic [*F*(4.21, 332.35) = 26.94, *p* < 0.001, η^2^ = 0.09, 95%–CI [0.03, 0.14]] and a significant interaction [*F*(3.53, 278.96) = 22.54, *p* < 0.001, η^2^ = 0.05, 95%–CI [0.00, 0.09]]. *Post-hoc* paired *t*-tests showed significant differences for all characteristics (all *p*-values < 0.001) except causality [*t*(79) = 1.96, *p* = 0.053] and speed [*t*(79) = 0.63, *p* = 0.533] with the robot in the multiple cues condition receiving higher ratings for all characteristics (see Figure 3A).

For selfhood-attribution data, the Bayesian analysis only provides decisive evidence for an effect of subscale (*BF*_10_ > 100, ±0%), but no conclusive evidence for cues (*BF*_10_ = 2.91, ±0%) and decisive evidence against an effect of the interaction (*BF*_01_ > 100, ±0%). In contrast, the ANOVA showed significant main effects of cues [*F*(1, 79) = 11.50, *p* = 0.001, η^2^ = 0.01, 95%–CI [0.00, 0.05]] and subscale [*F*(3.70, 292.19) = 155.67, *p* < 0.001, η^2^ = 0.50, 95%–CI [0.41, 0.55]], as well as a significant interaction [*F*(4.03, 318.20) = 2.53, *p* = 0.040, η^2^ = 0.00, 95%–CI [0.00, 0.01]]. Notably, in this experiment, the effect size of the main effect of the cue and the interaction is negligible (η^2^ < = 0.01, see Cohen, 1988). Overall, these results suggest that there are no substantial differences in selfhood-attribution between the single and multiple cues robot (see Figure 3B).

### 3.3 Experiment 3: multiple cues vs. equifinality

**Figure 4:**
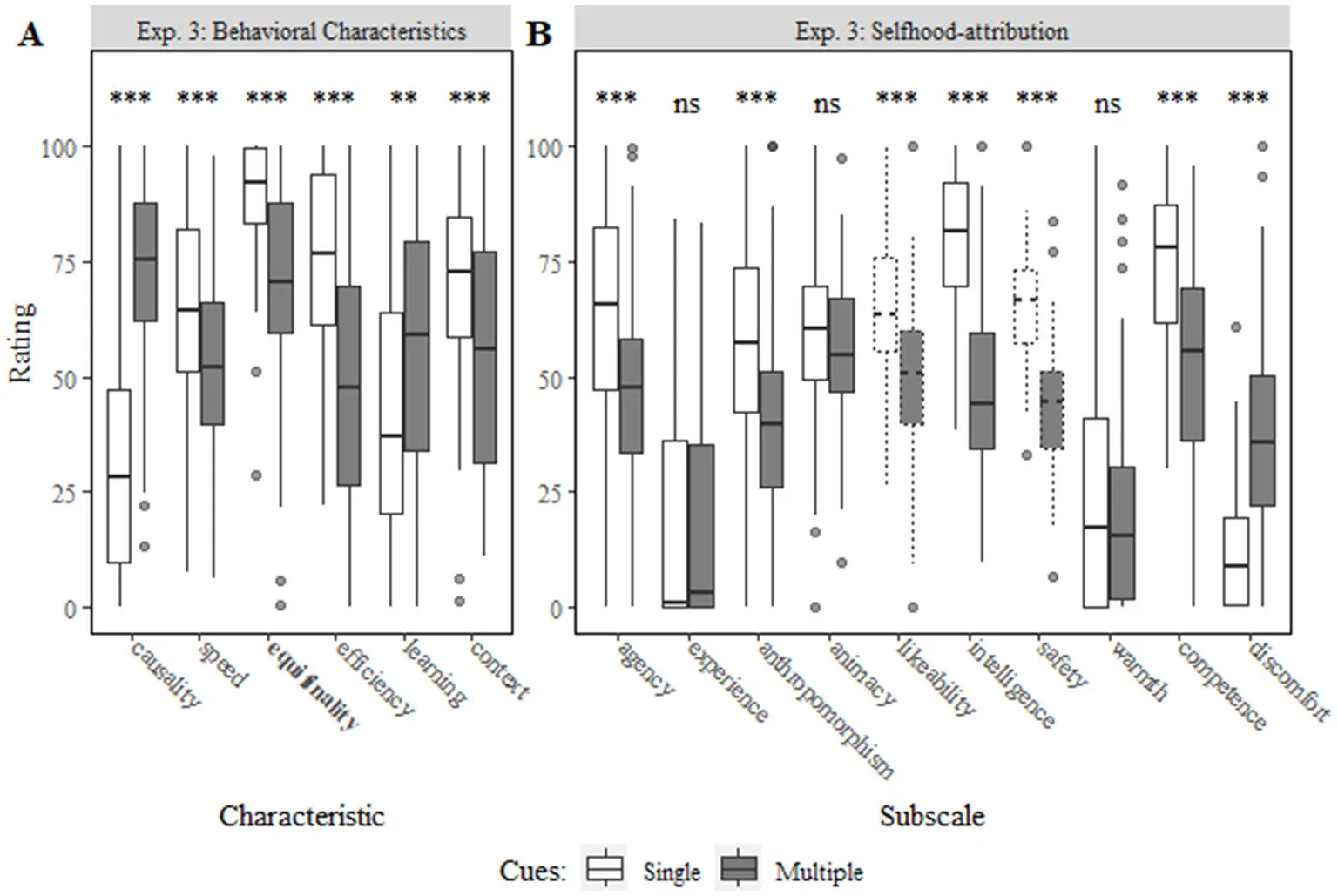
Results of experiment 3 (multiple cues vs. learning). **(A)** Results for the behavioral characteristics. **(B)** Results for the selfhood-attribution. Barplots printed with dotted lines are not considered critical for selfhood-attribution. Note, for the selfhood-attribution that while the frequentist ANOVA showed a significant interaction, the effect size was negligible (< 0.01), and the Bayesian analysis revealed decisive evidence against the interaction. Significance codes: *p* < 0.050*, *p* < 0.010**, *p* < 0.001***.

For the behavioral characteristics, the Bayesian analysis revealed revealed substantial evidence against a main effect of cues (*BF*_01_ = 3.84, ±0%) and decisive evidence for both the main effect of characteristic and, more interestingly, the interaction (both *BF*_10_ > 100, ±0%). Conversely, the ANOVA revealed a significant main effect of characteristic [*F*(3.77, 297.56) = 52.14, *p* < 0.001, η^2^ = 0.15, 95%–CI [0.08, 0.22]] and, more interestingly, a significant interaction [*F*(3.87, 305.57) = 50.47, *p* < 0.001, η^2^ = 0.19, 95%–CI [0.11, 0.26]]. *Post-hoc* paired *t*-tests showed significant differences for all characteristics (all *p*-values < = 0.005) with the robot in the single condition receiving higher ratings for all characteristics except causality and learning sensitivity, for which it was rated lower than the robot in the multiple cue condition (see Figure 4A).

The Bayesian analysis of the selfhood-attribution data revealed decisive evidence for both main effects and the interaction, as compared to the null model (all *BF*_10_ > 100, ±0%). Consistent with the results, the ANOVA showed significant main effects of cues [*F*(1, 79) = 39.46, *p* < 0.001, η^2^ = 0.07, 95%–CI [0.00, 0.17]] and subscale [*F*(3.98, 314.42) = 168.69, *p* < 0.001, η^2^ = 0.43, 95%–CI [0.34, 0.49]], and a significant interaction [*F*(4.14, 326.82) = 44.61, *p* < 0.001, η^2^ = 0.12, 95%–CI [0.06, 0.18]]. *Post-hoc* paired *t*-tests further revealed significant differences for all subscales except experience [*t*(79) = 0.07, *p* = 0.942], animacy [*t*(79) = 1.49, *p* = 0.141] and warmth [*t*(79) = 1.37, *p* = 0.175] (all other *p*-values < = 0.010). Ratings were higher for the robot in the single condition on all subscales with significant differences, except discomfort, where it was rated lower than the other robot (see Figure 4B).

### 3.4 Joint analysis

**Figure 5:**
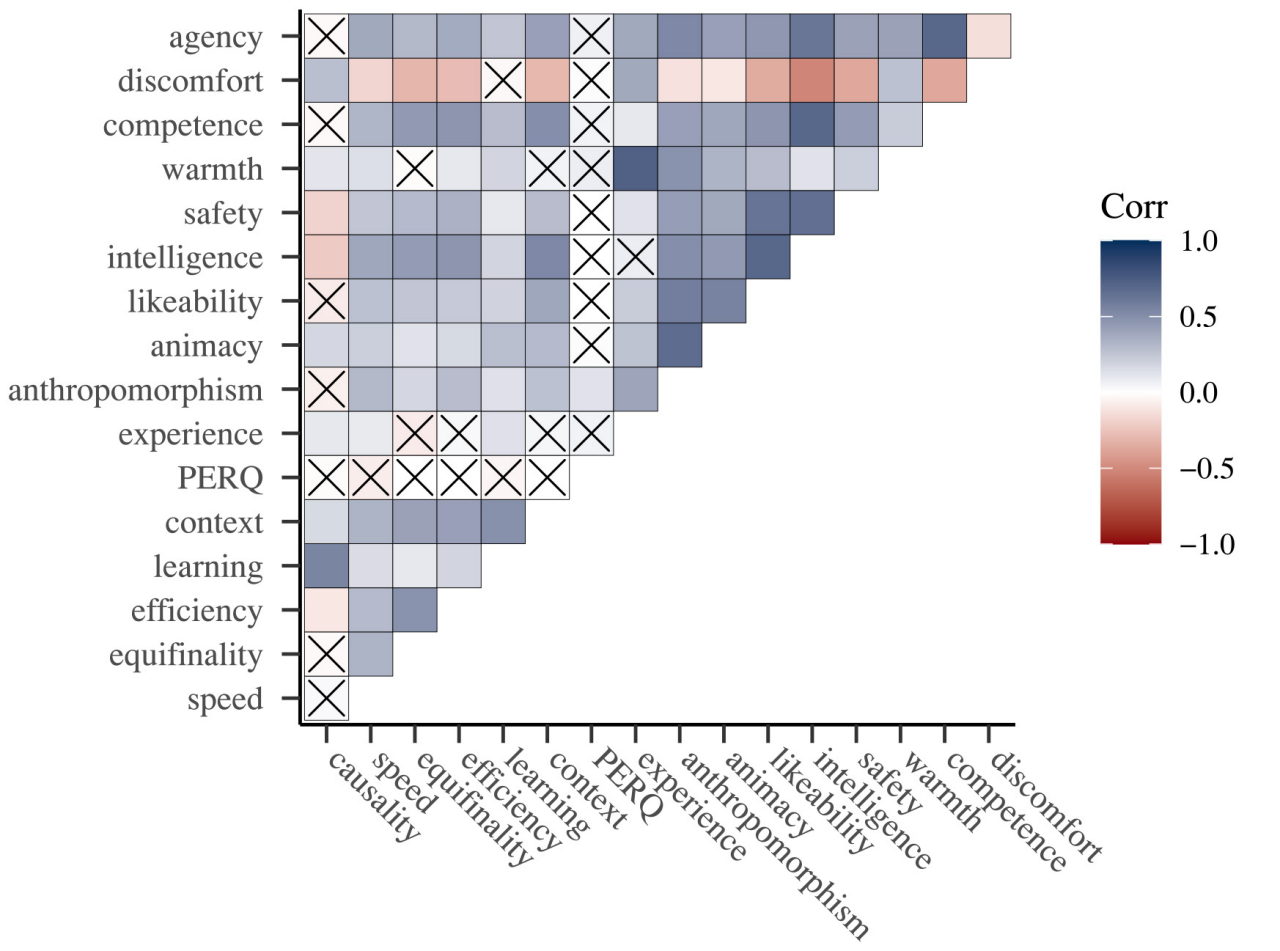
Correlation plot across all experiments. Crossed out correlations are not significant (*p* ≥ 0.050).

To identify commonalities across the 3 experiments, we calculated Pearson's correlation coefficients using pairwise deletion for ratings of the characteristics and selfhood-attribution subscales across all experiments. This analysis showed that a majority of characteristics and subscales have significant, positive correlations (65% of all correlation coefficients), with moderate coefficients accounting for 26% and strong coefficients accounting for 16% of all coefficients (see Figure 5 for visual representation).

Notably, for the subscale discomfort, almost all of the significant correlations are negative, which makes sense as this is the only negatively framed subscale. This subscale further shows an over proportionate number of non-significant correlations, suggesting that it is not a crucial dimension for selfhood-attribution. Further, it is interesting to see that causality is negatively correlated with perceived safety and intelligence. Causality reflects the agents' ability to impact or change their environment, and in our stimuli, which is operationalized by the robots pushing the black cubes or “crashing” into the white stationary cubes. Following, it is no big surprise that the robot pushing and crashing into cubes is perceived as less safe. Moreover, the repeated attempts at pushing white cubes, while necessary to show learning effects, most likely were interpreted as less intelligent behavior.

Finally, to assess the reliability of the modified measurement format, for each experiment and condition, we calculated Cronbach's alpha coefficients for the individual subscales. In 83% of cases, internal consistency reached acceptable or higher levels (α > 0.70). Notably, alpha values below 0.70 occurred only for the perceived safety and animacy subscales, with perceived safety not constituting a primary variable of interest in the present study.

### 3.5 Explorative analysis

To explore the effects of prior experience with robots on participants attribution pattern we fitted a linear mixed effects model with within-participant factors, cues, and subscales, as well as the between-participant factor PERQ (self-reported, ranging from 1 to 5) with the formula “*rating*~subscale*cue*PERQ+(1|*id*).” A comparisons of this full model with a reduced model excluding PERQ revealed a better model fit of the full model (*X*^2^(20) = 45.97, *p* < 0.001). A likelihood-ratio test of the full model suggests only significant evidence for an interaction of PERQ and subscale (*X*^2^(9) = 27.91, *p* < 0.001) and no significant evidence for either the main effect, interaction with cue, nor for the three-way interaction (all *p*-values >= 0.100).

**Figure 6:**
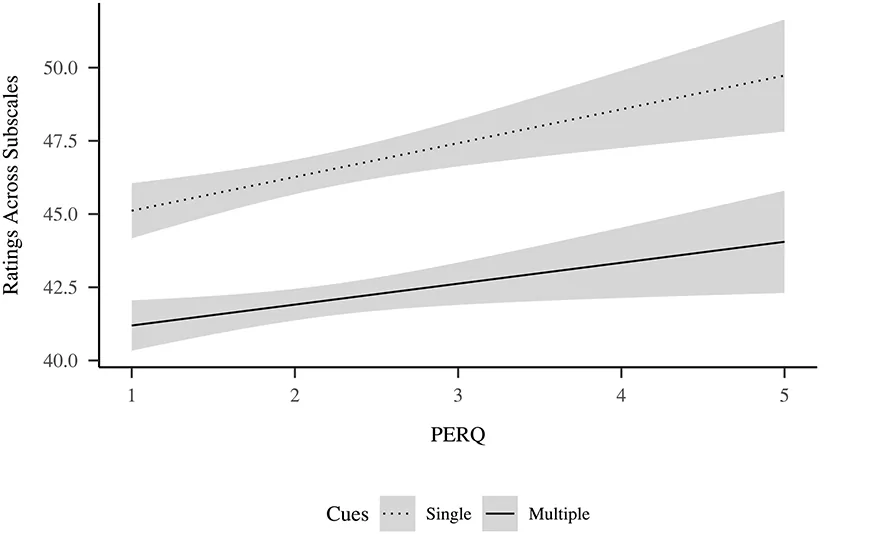
Selfhood-attribution based on prior experience with robots. GLM-fit of ratings across all selfhood-related subscales separated by cue number.

Descriptively, a trend is visible for an increase in the overall ratings of the robots with higher knowledge about robots (see Figure 6). This pattern is the same for both cue conditions, though it can be seen that across experiments, ratings for the single-cue robot are consistently higher for all participants. Which is interesting because previously we found that higher expertise with robots was linked to a more pronounced difference between the critical robot showing cues for a social characteristic and the control robot, suggesting the absence of the same characteristic (Nikolovska et al., 2024). In that study, only the critical robot was rated higher by participants with more prior experience with robots, whereas we found a reverse pattern for the control robot. This might suggest that while we did not expect to observe differences in selfhood-attribution in the present study, attributing more characteristics of an agent are simply linked with a higher attribution of selfhood to this agent. Conversely, participants might indeed have seen cues for self-related behavior in both robots, but simply showed a stronger attribution to the single cues robot, which will be discussed in the next section.

## 4 Discussion

We have previously shown that behaviorally cued characteristics relevant in selfhood-attribution tend to be over-generalized from cues that participants actually experience other self-related characteristics for which they do not experience cues, which we framed the *Pars-Pro-Toto* account (Pohl et al., 2026, 2025). It is important to clarify that this account is conceptually related to well-established phenomena such as the halo effect or illusory correlations. As we have expressed in our preceding studies, this account assumes that represented characteristics and the judgments they produce can extend beyond the information immediately available by drawing on previously learned associations among those characteristics. As a result, such a framework can generate not only the specific findings observed in this study, but also any form of halo effect. This implies that the model explains phenomena that extend well beyond the present observations. We suggest that in the context of selfhood-attribution, these associations are organized within a social-cognitive feature network. Within this network, a single salient behavioral signal acts as a gateway that activates a broader, pre-existing conceptual model of the agent as a “self”, even when evidence for other agentic traits is absent.

Inspired by this observation, we hypothesized that an agent showing cues for multiple characteristics will not be perceived differently from an agent showing only a single cues, as participants should over-generalize from this single cue to others anyway. The key question driving the present study was, therefore, whether selfhood-attribution would differ based on the number of behavioral cues presented by an agent. For this aim, across three experiments, we compared a non-humanoid robot agent suggesting the presence of multiple self-related characteristics (*multiple cues* condition)—namely, efficient behavior, learning sensitivity and equifinality (as identified in our previous experiments)—with a robot presenting behavioral cues for only one of these characteristics (*single cues* condition).

### 4.1 Summary and interpretation of results

In *Experiment 1*, we compared the robot in the multiple cues condition with a robot suggesting only the presence of efficiency. Here, two findings are noteworthy: First, contrary to our assumption, participants did perceive the two robots differently. Surprisingly, they rated the robot showing only cues for one characteristic *higher* regarding most of the behavioral characteristics, including equifinality, which was exhibited only by the other robot (in the multiple cues condition). This suggests that the over-generalization we observed previously, was *stronger* for the robot showing *fewer* cues. The second finding is that the selfhood-attribution was stronger for the robot in the single cues condition, which is consistent with our previous studies, that a stronger perception of self-related behavioral characteristics are linked to higher selfhood-attribution.

In *Experiment 2*, we compared the robot in the multiple cues condition with a robot exhibiting only cues for the single characteristic of learning sensitivity. Moreover, participants perceived the behavior of the two robots differently. Only this time, they perceived the multiple-cue robot to show *more* equifinality, efficient behavior, as well as learning and context sensitivity, than the single-cue robot. Conversely, they numerically correctly identified the manipulated characteristics in the multiple cues condition and extended this attribution to context sensitivity, but showed no evidence of over-generalization in the single cues condition. However, while the interaction was significant in the traditional ANOVA, the effect size was negligible, and the Bayesian approach revealed decisive evidence against this interaction. Accordingly, we tend to interpret this finding as the absence of meaningful differences between the two robots regarding selfhood-attribution.

In *Experiment 3*, we compared the robot with multiple cues condition with a robot showing only cues for equifinality. In this experiment, we replicated the findings of Experiment 1, as participants again rated the robot in the single cues condition higher for the majority of the behavioral characteristics, as well as on the scales assessing selfhood-attribution. Furthermore, this over-generalization included characteristics, such as efficiency, that were not manipulated in the single-cue robot.

### 4.2 Limitations

These results are constrained by the same limitations as those that apply to our previous studies. Specifically, the operationalization of our stimuli preclude the possibility of manipulating certain characteristics individually. For instance, learning sensitivity was demonstrated by the robot “learning” that only the black cubes are movable, however, for this purpose, the robot has to interact with the cubes and is thus also providing cues for the characteristic causality. In Experiment 3, this was also reflected in the participants' perception of the robots. Both learning sensitivity and causality were rated significantly higher in the same robot (the one in the multiple cues condition). However, this pattern is not observed in the other experiments. In Experiment 1, the robot exhibited multiple cues, including cues for the ability to learn, was not rated higher as more capable of learning; however, it was rated higher in regard to causality. Next, it is notable that in the two experiments where robust differences in attributions of selfhood were observed, the robot that was rated higher on the corresponding scales, and was also perceived as exhibiting fewer cues for causality. In the first experiment of our preceding study (Pohl et al., 2026), participants perceived one robot as causal and the other as context sensitive, with the latter receiving higher ratings for selfhood-attribution. In sum, these findings suggest that the operationalization of causality may lead to the interpretation of unintelligent or “clumsy” behavior not perceived as typical of a human-like, self-possessing agent. This is also reflected in the joint correlation analysis in the present study, which indicates a mix of weak positive and negative, as well as non-significant correlations between causality and the other variables. Furthermore, it is important to highlight that participants rated the robot with higher selfhood scores, also, for example, as moving with a more human-like speed than the other robot, despite the absence of any manipulation of speed. Overall, the Pars-Pro-Toto account of this over-generalization from observed to unobserved characteristics, thus, cannot be reduced to a mere confound in the stimulus material.

However, we do concur that movement complexity and collision behavior pose confounds in interpreting the selfhood-attribution data. The stronger attributions for simpler behaviors may reflect an observer's preference for more salient or less “noisy” agents rather than a pure effect of cue number. However, we argue that this reduction in “noise” and an increase in saliency are inherently linked to the reduction of cue quantity; a robot displaying multiple cues, such as learning through repeated “clumsy” interactions with its environment, necessarily presents a more complex and potentially ambiguous behavioral signal than a single-cue agent. From the perspective of our Pars-Pro-Toto account, the increased perceptual coherence of a single, salient cue likely facilitates the observer's ability to “bootstrap” a unified internal model of the agent more effectively than a multi-layered behavioral sequence. Thus, while the qualitative aspects of the movement differs, we believe our interpretations remain valuable as they highlight how perceptual clarity in minimal behavior serves as a powerful driver for the inference of a coherent self-concept. Still, future research should employ a factorial stimulus design that independently manipulates cue quantity and movement complexity to address this with certainty.

### 4.3 Theoretical implications

The data presented here demonstrate that our Pars-Pro-Toto account of self-attribution has more complex implications than we have previously considered. If individuals were to merely overgeneralize from the presented cues, which suggest capabilities of self-related characteristics, then they should have perceived the single and multiple cues condition robots more or less the same way. For the single condition, the cues presented for one characteristic would lead to the assumption of others, whereas for the multiple cues condition, there are actually multiple cues that can trigger the perception that it is capable of various self-related characteristics. However, this does not appear to be the case; rather, we observed a more pronounced shift in overall self-related attributions for the robot with fewer capabilities. What can be inferred about this manner in which individuals utilize their naïve concept of the self?

In light of the present data, it is intriguing to consider why individuals would ascribe a greater number of self-related attributes to an agent that displays a smaller set of cues typically associated with the concept of selfhood. It may be the case that the presentation of multiple behavioral cues are perceived as less salient than the consistent display of single cues associated with one specific characteristic. This is consistent with the literature on cognitive *bootstrapping* (the idea of developing new and complex knowledge from a limited starting set; see American Psychological Association, n.d.), particularly the program synthesis approach. In a study designed by Correa et al. (2025), participants were invited to engage in a programming game, with the aim of gaining insight into the hierarchical structure of action planning. The objective of this study was to devise a program that would move a virtual robot to a series of designated goal locations, with a monetary incentive to produce a program of minimal length. However, the researchers observed that participants preferred to write programs that reused components rather than minimizing the length of the program description. This form of bootstrapping has been proposed as a uniquely human capability relevant to our development (e.g., Carey, 2004). Studies that integrate cognitive modeling with behavioral experiments has demonstrated that this concept can explain a range of phenomena, including causal learning (Zhao et al., 2024) and visual learning (Zhou et al., 2024), as well as the use of prototypical geometric shapes to create complex line drawings (Sablé-Meyer et al., 2022). In our present experiments, the repeated presentation of one specific type of cue in the single cues condition may have initiated a similar bootstrapping process, resulting in the formation of a model of an agent that can be best described as having self- or self-related characteristics. Moreover, the reuse of a single characteristic to solve the “problem” of reaching a goal might more closely reflect a human-directed approach, thus resulting in the single cues condition robot appearing more human-like and, by extension, more likely to possess a self. We present this cognitive bootstrapping interpretation as a hypothesis for future modeling rather than a definitive developmental claim. In this context, we suggest that the consistent display of a single cue may trigger a “perceptual bootstrapping” process in the observer. By observing the repeated and successful reuse of a specific skill (like efficiency), the observer may synthesize a mental model of the robot as a structured, human-like agent—paralleling the program synthesis approach, where simplicity and reuse are prioritized over complexity. Future research may thus try to model the presented data with a program synthesis approach to further explore the possibility of a bootstrapping process in selfhood-attribution.

Furthermore, our findings can be interpreted through the lens of coherence-based inference theories (e.g., Thagard, 1989) similar to Brunswik's (1955) probabilistic functionalism that inspired our research. The coherence theories suggest that human judgement is guided by a “parallel constraint satisfaction” process (e.g., Glöckner et al., 2014) aimed at creating a “best-fit” interpretation of ambiguous data. According to these frameworks, individuals tend to favor interpretations that maximize internal consistency and minimize conflicting signals (see also Thagard and Millgram, 1995). In our experiments, the single-cue robots (displaying direct, consistent behavioral signals) likely provided a highly coherent and “readable” signal for the observer. In contrast, the multiple-cues robot presented a “noisier” signal that may have introduced conflicting information, thereby hindering the observer's ability to project a unified, agentic internal state. This suggests that the “less is more” account may be driven by the cognitive system's preference for informational clarity and perceptual fluency over sheer behavioral complexity.

Finally, the results of this study have a practical implication for the field of human-robot interaction. This suggests that, contrary to intuitive expectations, the design of simpler, more consistent behavioral patterns may be more effective in enhancing perceived human-likeness than the implementation of more complex, multi-cue behaviors.

### 4.4 Conclusion

In summary, our study provides evidence that the Pars-Pro-Toto account of self-attribution is missing the complete picture in the description of merely an over-generalization based on a single cue; rather, we observed the tendency that there is a preference to over-generalize agents showing simple behavior. First, we demonstrate that our previous findings, which indicated a tendency for individuals to over-generalize attributed self-relevant characteristics from cues for any single characteristic, remain valid. Furthermore, the comparison with an agent, which exhibited cues for multiple characteristics, demonstrated across two of the three experiments involve simple behavior displaying behavioral cues for a single characteristic exert a stronger influence on the attribution of selfhood than complex behavior, suggesting the capacity for several characteristics. In the remaining experiment, we did not observe conclusive differences in selfhood-attribution between conditions.

A possible explanation is that the single cue condition presents more salient, or less “noisy”, behavioral cues relevant to selfhood. Under the lens of probabilistic functionalism or coherence-based inference theories, this would explain a preference for perceptual fluency. Moreover, we speculate that the over-generalization may be understood as a result of cognitive bootstrapping, the reuse of knowledge to form complex ideas that is a uniquely human phenomenon, suggesting it as a potentially fruitful framework for future (modeling) studies to investigate how the perceived reuse of simple actions might trigger the attribution of selfhood.

Overall, regardless of whether a self exists and how it is defined scientifically, individuals seem to construct a *naïve* concept of the self to interpret the behavior of other agents. Therefore, we propose that research should increase the focus on the self as a concept in social interaction rather than on the question of what the “real” self might be. Our evidence supports the approach that the self is primarily a social function relevant in human interaction, which should be investigated as such.

## Data Availability

The datasets presented in this study can be found in online repositories. The names of the repository/repositories and accession number(s) can be found below: osf.io/v62sg.

## Footnotes

1 Notably, the manipulation check scale includes cues that were not directly related to the experiments reported here, but that were found to be relevant in the attribution of selfhood in our previous studies.
